# THE MASON CARES RANDOMIZED STUDY: ASSESSING CAREGIVER BURDEN FOLLOWING A STRESS MANAGEMENT PROGRAM

**DOI:** 10.1093/geroni/igac059.1405

**Published:** 2022-12-20

**Authors:** Gilbert Gimm, Harveen Pantleay, Emily Ihara, Megumi Inoue, Shannon Layman, Catherine Tompkins

**Affiliations:** George Mason University, Vienna, Virginia, United States; George Mason University, Fairfax, Virginia, United States; George Mason University, Fairfax, Virginia, United States; George Mason University, Fairfax, Virginia, United States; George Mason University, Fairfax, Virginia, United States; George Mason University, Fairfax, Virginia, United States

## Abstract

**OBJECTIVE:**

The Mason CARES study examines the impact of a virtual stress management program and personalized music intervention on the stress of family caregivers of older adults with dementia. This study presents early findings on 31 participants (Cohort 1) who enrolled in January 2022.

**METHODS:**

Using primary data, we analyzed caregiver stress levels among 31 participants in Weeks 1 and 5 of a stress management program. Caregiver stress was measured using the Zarit Burden Interview (ZBI) score with a range from 0 to 48 (Bédard, 2001). Higher values (ZBI >=17.0) are associated with higher levels of stress.

**RESULTS:**

Among the 31 caregivers, 51.6% were spouses and 48.4% were non-spouses (e.g., adult children, nephew, etc.). While mean ZBI scores in Week 1 were similar for spouses and non-spouses (24.2 vs. 24.9), caregiver stress was higher when older adults had advanced versus moderate dementia (26.2 vs. 23.2). Between Weeks 1 and 5, spouses reported a greater decrease in stress (-2.6 vs. -2.2) compared to non-spouses. However, caregivers of older adults with advanced dementia experienced less of an improvement (-1.5 vs. -3.0).

**CONCLUSION:**

Findings show that all caregivers had high stress levels in Week 1 (ZBI score of 17 or more) with no difference between spouses and non-spouses. Between Week 1 and 5, spouses reported greater stress improvement than non-spouses. However, caregivers of older adults with advanced dementia had less improvement in stress. Future research will examine caregiver stress levels after random assignment to a Phase 2 music intervention group and control group.

